# Supplementary material for: Intercropping of Oats with Vetch Conducts to Improve Soil Bacteriome Diversity and Structure
Source: Microorganisms. 2025 Apr 24;13(5):977. doi: 10.3390/microorganisms13050977 (PMC12114406; doi:10.3390/microorganisms13050977)

Supplementary Figure S1.

Rarefaction curves of bacterial diversity (before sowing: S1-bulk soil; ripening phase: S2-bulk soil, S3-oats rhizosphere, S4-intercropping rhizosphere; 3 months after green manure: S5-intercripping field, S6-bulk soil).

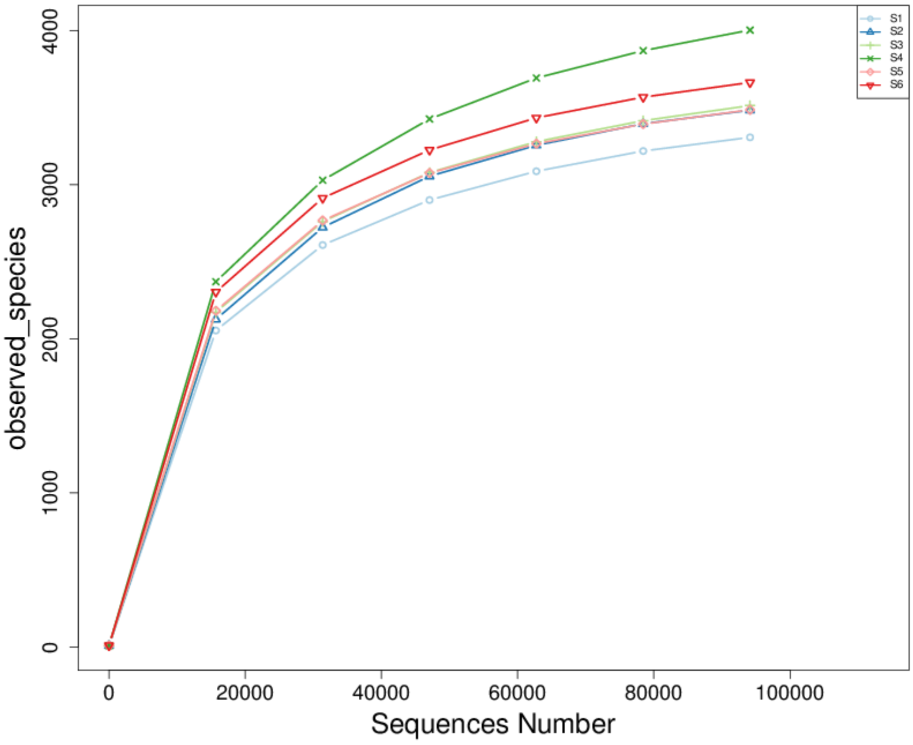

Supplement: Supplementary file 1 [file microorganisms-13-00977-s001.zip › microorganisms-3578337-supplementary.pdf]
